# Use of Whole Genome Sequencing Data for a First in Silico Specificity Evaluation of the RT-qPCR Assays Used for SARS-CoV-2 Detection

**DOI:** 10.3390/ijms21155585

**Published:** 2020-08-04

**Authors:** Mathieu Gand, Kevin Vanneste, Isabelle Thomas, Steven Van Gucht, Arnaud Capron, Philippe Herman, Nancy H. C. Roosens, Sigrid C. J. De Keersmaecker

**Affiliations:** 1 Transversal activities in Applied Genomics, Sciensano, J. Wytsmanstraat 14, B-1050 Brussels, Belgium; mathieu.gand@sciensano.be (M.G.); kevin.vanneste@sciensano.be (K.V.); Nancy.Roosens@sciensano.be (N.H.C.R.); 2 Viral Diseases, Sciensano, J. Wytsmanstraat 14, B-1050 Brussels, Belgium; Isabelle.Thomas@sciensano.be (I.T.); Steven.VanGucht@sciensano.be (S.V.G.); 3 Quality of Laboratories, Sciensano, J. Wytsmanstraat 14, B-1050 Brussels, Belgium; Arnaud.Capron@sciensano.be; 4 Expertise and Service Provision, Sciensano, J. Wytsmanstraat 14, B-1050 Brussels, Belgium; Philippe.Herman@sciensano.be

**Keywords:** SARS-CoV-2, COVID-19, detection, diagnosis, RT-qPCR, in silico specificity evaluation, bioinformatics tool, WGS data, mismatches, primers and probes

## Abstract

The current COronaVIrus Disease 2019 (COVID-19) pandemic started in December 2019. COVID-19 cases are confirmed by the detection of SARS-CoV-2 RNA in biological samples by RT-qPCR. However, limited numbers of SARS-CoV-2 genomes were available when the first RT-qPCR methods were developed in January 2020 for initial in silico specificity evaluation and to verify whether the targeted loci are highly conserved. Now that more whole genome data have become available, we used the bioinformatics tool SCREENED and a total of 4755 publicly available SARS-CoV-2 genomes, downloaded at two different time points, to evaluate the specificity of 12 RT-qPCR tests (consisting of a total of 30 primers and probe sets) used for SARS-CoV-2 detection and the impact of the virus’ genetic evolution on four of them. The exclusivity of these methods was also assessed using the human reference genome and 2624 closely related other respiratory viral genomes. The specificity of the assays was generally good and stable over time. An exception is the first method developed by the China Center for Disease Control and prevention (CDC), which exhibits three primer mismatches present in 358 SARS-CoV-2 genomes sequenced mainly in Europe from February 2020 onwards. The best results were obtained for the assay of Chan et al. (2020) targeting the gene coding for the spiking protein (S). This demonstrates that our user-friendly strategy can be used for a first in silico specificity evaluation of future RT-qPCR tests, as well as verifying that the former methods are still capable of detecting circulating SARS-CoV-2 variants.

## 1. Introduction 

In December 2019, an unusual and increasing number of pneumonia cases from unknown origin were reported in Wuhan, a city located in the province of Hubei, China. The typical signs of illness ranged from mild or absent symptoms to fever, cough, sore throat, loss of smell and taste, headache, fatigue, myalgia, and breathlessness, and were potentially life threatening. Therefore, an admission in intensive care units was occasionally required, especially for at-risk populations such as elderly patients and patients suffering from co-morbidities [1,2]. The responsible agent of these cases was identified as a novel coronavirus belonging to the *Coronaviridae* family, *Betacoronavirus* genus, and *Sarbecovirus* subgenus. This new virus, named Severe Acute Respiratory Syndrome Coronavirus 2 (SARS-CoV-2), initially 2019 novel Coronavirus (2019-nCoV), probably originated from bats, as its genome showed between 87% and 96% similarity with genomes of bat coronaviruses [3,4,5]. It is also suspected that SARS-CoV-2 jumped from bats to a human host through an intermediate animal vector, such as a pangolin, civet, or another animal species traded as living organisms at the Huanan wholesale seafood market of Wuhan. Indeed, coronaviruses isolated previously from such animals also show a close genomic similarity with the novel human coronavirus [5,6], although this hypothesis needs further investigation to be validated [7]. Outbreaks due to other zoonotic agents from the *Coronaviridae* family, and related to SARS, were previously observed in 1965 (HCoV-229E, still circulating each winter season), at the end of 2002 (SARS-CoV) and in 2012 (MERS-CoV) [8]. Genomic comparisons between SARS-CoV (also belonging to the subgenus *Sarbecovirus*) and SARS-CoV-2 showed a more than 70% similarity [1]. Since its first occurrence in December 2019 in China, the SARS-CoV-2 outbreak, leading to COronaVIrus Disease 2019 (COVID-19), spread around the world. Therefore, on 11th March 2020 the World Health Organization (WHO) declared that this outbreak had reached a pandemic state. At the time of writing of this article (24/06/2020), 9,263,570 confirmed cases in 188 countries have been reported, of which 477,584 resulted in death [9,10].

During a pandemic such as the current one caused by SARS-CoV-2, one of the major concerns for public health authorities is the ability of diagnostic laboratories to rapidly and accurately identify the presence of the virus in the population. This is particularly important, firstly, to provide medical surveillance and assistance when required, and secondly to isolate infected patients in quarantine with the aim of limiting the spread of this highly contagious virus to non-infected people in order to reduce the risk of further dispersion, especially within at-risk groups. In addition to the presence of certain symptoms (as described in the previous paragraph), SARS-CoV-2 infection can be diagnosed through the detection of lung lesions by Computed Tomography (CT), by the detection of specific antibodies (IgM and IgG), produced in reaction to the infection, by immunological assays (such as Enzyme Linked ImmunoSorbent Assay (ELISA) and rapid antibody tests) to assess seroconversion, and by the determination of the presence of the virus itself by nucleic acid detection methods (such as Reverse Transcription real-time PCR (RT-qPCR)) or by using sequencing technologies. Chest CT scans provide a good clinical indication of pneumonia and are complementary to other methods [11,12,13], but are not specific to SARS-CoV-2. Sequencing technologies are accurate and provide complete genome data but are time-consuming and unsuitable for rapid diagnostics. They are mostly used after a positive RT-qPCR result. RT-qPCR tests are indeed useful at the early disease stage, when the virus can be detected in the respiratory tract. Immunological assays are more adapted for a later disease stage when IgM antibodies are detectable in the blood stream 3 to 6 days after the first onset of symptoms, while in parallel the viral load starts to decrease in the body and cannot be detected by RT-qPCR anymore [14,15,16,17]. Therefore, RT-qPCR was rapidly adopted as the primary diagnostic test to quickly detect SARS-CoV-2 and to take the necessary medical and quarantining measures in time. Nevertheless, a non-negligible rate of false negative results were reported when using this technique [18]. One possible explanation is that the viral RNA concentration can be too low in the sample to be properly detected by RT-qPCR methods. Indeed, the viral load can vary depending on the source (nasopharyngeal, oropharyngeal, bronchoalveolar lavage, sputum, saliva, or feces) and quality of the sample, as well as the sampling time (too early before the symptoms’ onset or too late during the symptoms’ relief) [13,19,20]. Another explanation for the false negative rate of RT-qPCR results could be the natural ability of viruses to genetically evolve [21,22]. Indeed, as qPCR methods are based on the use of primers and probes that must specifically anneal to their complementary template sequence, substitutions or deletions in the viral genome can have an impact on the test outcome. Depending on the location and number of mismatches between the primer/probe sequences and their template, the consequences on the qPCR signal can vary. For instance, while one mismatch can have a minor effect, two or three mismatches can potentially lower the sensitivity of the method as well as increase the quantification cycle (Cq) value, and more than three mismatches can cause a total reaction failure. Additionally, it is known that a mismatch located at the first nucleotides of the 3′ end of the primers has a large negative impact [23,24,25,26].

In the early stage of the pandemic, as soon as some SARS-CoV-2 genomes were fully sequenced by Whole Genome Sequencing (WGS), RT-qPCR assays were developed rapidly in January 2020, responding to the need to detect SARS-CoV-2 RNA in the biological samples of people with the suspicion of infection. Some of these methods were recommended by the WHO in their technical guidance for laboratory testing published the 24th of January 2020 [27]. However, as these methods were developed at an early stage of the pandemic as there was no other option, it was not possible to clearly evaluate what were the most conserved genomic regions of SARS-CoV-2 ideal for primers and probe design. Additionally, too few SARS-CoV-2 genome sequences were available at that time to perform an exhaustive in silico specificity evaluation of the designed primers and probe sets. Finally, although control samples became quickly available, the scientific team developing these RT-qPCR assays did not have a large collection of SARS-CoV-2 strains to perform extensive valid inclusivity tests in the wet lab. Moreover, some of these tests were later shown to have large differences in performance [28,29]. Since the early development of the WHO recommended tests, several additional RT-qPCR assays were published [30,31,32,33,34]. In parallel, thousands of SARS-CoV-2 genomes became publicly available and continue to be released. Therefore, it is relevant now to use all genome data available to evaluate the current in silico specificity of the RT-qPCR tests, including those developed at the onset of the pandemic. Indeed, regarding the ability of viruses in general to genetically evolve, the genomic regions targeted by viral RT-qPCR tests must be evaluated periodically to assess whether they are stable in time or whether their mutation rate could lead to false negative results in the future. Although other studies have already evaluated mismatches in primers and probes used for SARS-CoV-2 detection, they have often only incorporated a limited number of genome sequences or RT-qPCR assays, or only evaluated the situation at a single time point [28,35,36,37,38]. Additionally, such systematic investigations present a substantial bottleneck for routine diagnostic laboratories, which often do not have access to the required bioinformatics expertise and/or resources, especially when considering the intricacies encountered in the proper design of primers and probes [39]. The manual alignment of primers and probes to thousands of individual genomes is furthermore highly time-consuming. To overcome these limitations, we previously developed an open-access bioinformatics tool named SCREENED (polymeraSe Chain Reaction Evaluation through largE-scale miNing of gEnomic Data [39]), enabling user-friendly investigations of mismatches between the primers and probes employed in routine RT-qPCR methods, and large amounts of whole genome data, evaluating for each genome in silico the generation of a theoretical RT-qPCR signal. This method has already been successfully used for the evaluation of RT-qPCR tests used for Dengue virus detection [39], as well as for the Zika and Chikungunya viruses [40].

In the current study, we used SCREENED to perform a large-scale in silico specificity evaluation of 30 primers and probes sets (i.e., assays), part of 12 RT-qPCR tests developed for the detection of SARS-CoV-2. From a dataset of 2569 unique SARS-CoV-2 genomes obtained after the clustering of 3590 publicly available genomes from different databases, SCREENED extracted the genomic regions targeted by the evaluated RT-qPCR assays and assessed whether primers and probes could properly anneal, theoretically resulting in correct virus detection. From this, the inclusivity of each assay was determined. Additionally, the same analysis was performed using 2423 representative unique genomes belonging to other coronaviruses and other respiratory viruses in addition to the human reference genome, with the aim of also evaluating the exclusivity of each assay. Finally, the inclusivity of four RT-qPCR tests was investigated again, with a new batch of 1165 SARS-CoV-2 genomes downloaded one month after the first download, corresponding to 968 representative unique sequences, with the aim of assessing the impact of the viral genetic evolution after this period of time.

## 2. Results

### 2.1. Overview of RT-qPCR Assays for SARS-CoV-2 Detection

**Figure 1:**
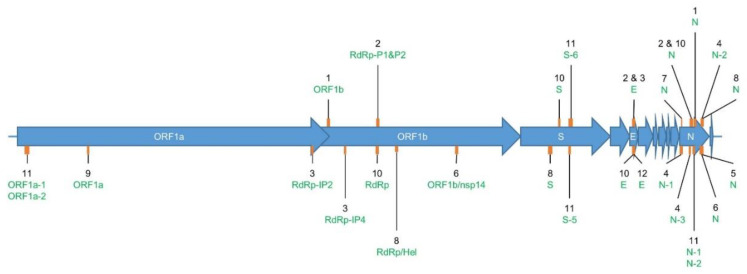
Location of the sequence amplified by each evaluated primer set in Table 2. Genome (NC_045512.2). The SARS-CoV-2 genome (~29,000 nt) is composed of genes coding for structural proteins, such as the Spike protein (S), Envelope protein (E), and Nucleocapsid protein (N); and non-structural proteins located in the Open Reading Frame 1ab (ORF1ab), such as RNA-dependent RNA polymerase (RdRp), Helicase (H), and non-structural protein 14 (nsp14). The orange rectangles in the figure show the approximate size and location in these genes of the target sequence that is amplified by each of the evaluated primer sets. The corresponding assay reference number is indicated in black, and its targeted gene in green (Table 1). Two labels connected to the same orange rectangle indicate that the targeted amplified sequences are overlapping. The exact starting point of each of the forward primers and the length of their corresponding amplicons are available in Table 1.

In the current article, a RT-qPCR test, as developed by a specific institute or scientific team (Table 1), was considered as composed of one or several singleplex assays—i.e., primers and probe sets. Twenty-four primers and probe sets using TaqMan technology and six primer sets using SYBR Green technology, corresponding to a total of 12 RT-qPCR tests, were collected for evaluation (Table 1). The targets of the 12 RT-qPCR tests are located in the SARS-CoV-2 genes coding for the envelope protein (E), the nucleocapsid protein (N), the spiking protein (S), and non-structural proteins located in the Open Reading Frame 1ab (ORF1ab), such as the RNA-dependent RNA polymerase (RdRp)/helicase (Hel) and the non-structural protein 14 (nsp14) (Figure 1). Seven RT-qPCR tests originated from the WHO technical guidance published for laboratory testing of SARS-CoV-2 in humans [27] and were developed by (in no specific order) the Chinese Center for Disease Control and prevention (CDC) (Assays_1 (ORF1b and N)), the German Charité Hospital (Assays_2 (RdRp-P1, RdRp-P2, E, and N)), the French Institut Pasteur (Assays_3 (RdRp-IP2, RdRp-IP4, and E)), the US CDC (Assays_4 (N-1, N-2, and N-3)), the Japanese National Institute for Infectious Diseases (NIID) (Assay_5_N), the Hong-Kongese Faculty of Medicine HKU Med (Assays_6 (RdRp/nsp14 and N)), and the Thai National Institute of Health (NIH) (Assay_7_N). Other assays (5) were retrieved from the literature or from a working document of the European Commission inventorying the diagnostic tests developed for COVID-19 [34]—i.e., Assays_8 (RdRp/Hel, S, and N)), Assays_9_ORF1a, Assays_10 (RdRp, S, E, and N), Assays_11 (N-1, N-2, ORF1a-3, ORF1a-4, S-5, and S-6), and Assay_12_E. Commercial kits could not be included in this study because, although it is most likely they are based on previously published protocols, the exact sequence of the primer and probe sets used in these tests is typically not communicated. Among all assays, the N gene was targeted most frequently. The annealing temperatures recommended by the authors of the assays for using their corresponding primers and probe sets was equal to or above 55 °C for all the assays, but for Assays_1 (ORF1b and N), Assays_10 (RdRp, S, E, and N), and Assays_11 (N-1, N-2, ORF1a-3, ORF1a-4, S-5, and S-6), this information was not communicated (Table 1).

To avoid incorrect diagnosis and improve specificity, most RT-qPCR tests evaluated in this study included multiple targets within the SARS-CoV-2 genome. Therefore, the results of some RT-qPCR tests must be interpreted taking into consideration the combination of multiple detected targets—i.e., assays. To clarify this, the authors of these tests gave specific recommendations, as described in Table 1. For instance, some primers and probe sets, such as the ones in Assay_2_E and Assay_3_E, can detect members of the *Sarbecovirus* subgenus and can be used for a first screening. Then, one or multiple other gene(s) (Assay_2_RdRp-P2, Assay_3_RdRp-IP2, and Assay_3_RdRp-IP4) should be targeted for confirming the presence of SARS-CoV-2 using more specific primers and probe sets for this virus. The detection of multiple SARS-CoV-2 specific targets, as seen for Assays_3 (RdRp-IP2 and RdRp-IP4), Assays_4 (N-1, N-2, and N-3), Assays_8 (RdRp/Hel, S, and N), Assays_10 (RdRp, S, E, and N), and Assays_11 (N-1, N-2, ORF1a-3, ORF1a-4, S-5, and S-6), allows to deal with the diversity and/or potential evolution of the virus. In general, for multi-target tests the following rules are applied for result interpretation: if all the targets of a RT-qPCR test are detected, the patient is a confirmed positive case for SARS-CoV-2; if none of the targets are detected, the absence of the virus is confirmed; and if there exists a mix of positive and negative results for the targeted genes, the test is inconclusive and has to be repeated or further investigation needs to be performed. The authors of Assays_10 (RdRp, S, E, and N) developed their test mainly for the rapid and cost-effective discrimination of non-infected people—i.e., negative for the four targets, who then know they cannot contaminate other people and can continue their daily life without quarantining [31]. 

Concerning Assay_2_N and Assay_4_N-3, even though their corresponding primers and probes were not included in the final protocol version of these tests [27], they were still evaluated in this study. Assay_2_RdRp-P1 and Assay_2_RdRp-P2 use identical primers but with different probes for the detection of the RdRp gene: P1 is pan-*Sarbecovirus*-specific, while P2 is strictly specific to SARS-CoV-2. Additionally, Assay_2_E and Assay_3_E use the same primers and probe to target E gene. Finally, Assays_11 (N-1, N-2, ORF1a-3, ORF1a-4, S-5, and S-6) consists of two primer sets (SYBR Green) and four primer and probe sets (TaqMan), targeting a similar locus of the genes N, ORF1a, and S (Figure 1 and Table 1). 

### 2.2. Determination of RT-qPCR Assay SARS-CoV-2 Inclusivity 

**Figure 2:**
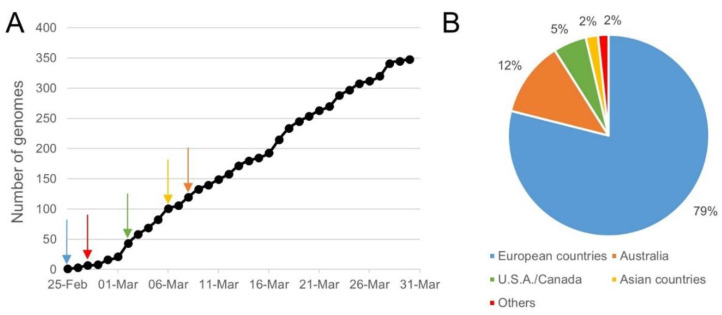
Sampling time and location for genomes that showed three mismatches in the sequence of Assay_1_N′s forward primer. Three mismatches between the forward primer sequence of Assay_1_N targeting N gene and 358 SARS-CoV-2 genomes were retrieved by SCREENED. Part (**A**) of the figure shows the occurrence of these genomes over time since the 25th of February 2020, and the arrows represent their first apparition in each continent according to the color legend in Part B. Ten of the 358 genomes with the described mismatches were not included in this figure, as their time of collection was not available. Part (**B**) of the figure shows the location where these genomes were collected. One of the 358 genomes with the described mismatches was not included in this figure, as its location was not communicated.

A total of 3590 SARS-CoV-2 genomes, sequenced from samples collected before the 7th of April 2020, were downloaded from the GISAID EpiCoV database, the NCBI Virus database, and the CNGB hCoV-19 database. After removing low-quality genomes and the clustering of identical genome sequences using CD-HIT, 2569 SARS-CoV-2 unique representative genomes were obtained and used in SCREENED for evaluating the inclusivity of the 12 RT-qPCR tests. The obtained results are listed in Table 2. Of a total of 30 different primers and probe sets evaluated, 13 were 100% inclusive, 16 showed an inclusivity of above 99.50%, and one showed an inclusivity of 86.03%. This last result concerned Assay_1_N of the China CDC, for which one substitution (A instead of G) and three substitutions (AAC instead of GGG) were identified at position 19 and position 1 of the reverse and forward primer sequences, and this for 1 and 358 genomes, respectively. The 358 genomes with three substitutions in their N gene were sequenced from samples collected between the 25th of February 2020 and the 30th of March 2020, mostly outside Asia—i.e., 281 in European countries (79%), 43 in Australia (12%), and 19 in USA/Canada (5%) (Figure 2). For these samples, SCREENED determined that Assay_1_ORF1b gave a theoretical positive result, while Assay_1_N was negative. Even if no specific recommendations were communicated by the China CDC for the overall interpretation of their RT-qPCR test, nor for the intended specificity of its two targets (strictly specific to SARS-CoV-2 or to *Sarbecovirus*), it is assumed that the overall result of this assay would be inconclusive for these 358 samples. 

**Figure 3:**
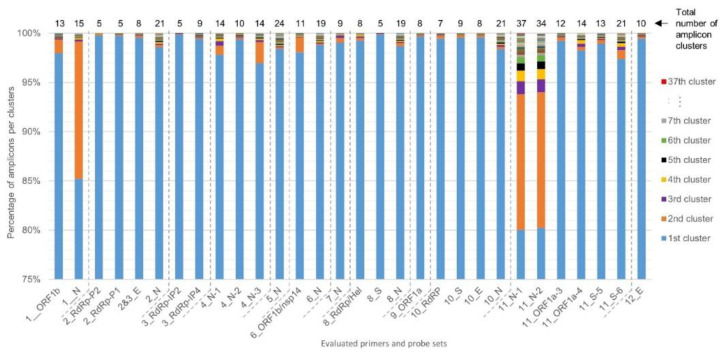
Diversity in the SARS-CoV-2 genomes of the target sequences amplified by the evaluated primer and probe sets. Clustering of the targeted genomic sequences amplified by the 30 evaluated primers was performed by SCREENED for each of the 12 RT-qPCR assays. The present chart shows the repartition of the amplicons from each genome in their sequence identity clusters (i.e., a set of targeted amplicons exhibiting exactly the same sequence), illustrating the overall sequence diversity according to the color key on the right of the figure for all primer and probe sets. For the majority of the assays, more than 97% of the amplicons were clustered in one large cluster. For Assay_1_N, Assay_11_N-1, and Assay_11_N-2, a second large cluster containing ~14% of the amplicons emerged. A varying amount of other clusters is present for the different methods, containing however only a very limited number of amplicons. Note that the y-axis, presenting the percentage of amplicons per cluster, starts at 75% to allow better the visual interpretation of amplicon diversity, since the first 75% always belongs to the first large cluster per primer and probe set.

For each of the 30 primers and probe sets tested, SCREENED was also used to perform clustering of the genomic sequences that are amplified during the RT-qPCR assays. The diversity among amplicons is presented in Figure 3. For most primers and probe sets, more than 97% of their corresponding retrieved amplicon sequences could be clustered together in one large cluster of identical amplicons, except for Assay_1_N, Assay_11_N-1, and Assay_11_N-2, which all three target the N gene for specific SARS-CoV-2 detection. The sequences amplified by Assay_11_N-1 and Assay_11_N-2 are very similar and have an overlap of 36 nucleotides with the amplicon sequence of Assay_1_N, meaning that they all target regions in a close vicinity in the N gene (Table 1). For Assay_1_N, 85% of the N amplicons clustered together and 14% were contained in a second large cluster consisting of 358 genomes, corresponding to those showing three substitutions (AAC instead of GGG) in the forward primer sequence described previously. An even more marked diversity was observed for Assay_11_N-1 and Assay_11_N-2, with, besides a second large cluster, several additional smaller clusters (Figure 3). Interestingly, despite showing the largest amplicon diversity, the inclusivity of Assay_11_N-1 and Assay_11_N-2 was determined to be above 99% (Table 2). After checking the alignments of the representative amplicon cluster sequences of Assay_11_N-1 and Assay_11_N-2 with their corresponding primers in MEGA X, this could be explained by the fact that the numerous SNPs responsible for the diversity of the genomic region targeted by these two assays are located in the center of the amplicon and not in the SYBR Green primers sequence (data not shown). In contrast to Assay_1_N, Assay_11_N-1, and Assay_11_N-2, Assay_8_S showed the smallest number of amplicon clusters (5), with more than 99% of the amplicon sequences contained in one main cluster. The inspection of the alignment of primers and probe of Assay_8_S with the representative sequences of these clusters indicated that only two mismatches were detected, one in the probe and one in the reverse primer sequence, each in one cluster containing only one genome, and no mismatches were detected in the forward primer sequence (data not shown). 

As a complementary approach to the mismatch criteria defined in SCREENED in agreement with the EU guidelines [34], sequence alignments of the amplicon clusters’ representative unique sequences were performed with their corresponding primers and probe sequences to investigate if some additional SNPs were present in a high number of genomes. A SNP was present in the sequence of the reverse primers of Assay_2_RdRp-P1&P2, Assay_5_N, Assay_8_RdRp/Hel, and Assay_10_E in unexpectedly all or almost all of the clusters—i.e., in more than 99% of the analyzed genomes. Similarly, two SNPs in the sequence of probe P1 of the Assay_2_RdRp-P1 were present in almost all the genomes (Table 3).

### 2.3. Determination of RT-qPCR Assay Exclusivity 

For exclusivity testing, 2624 non-SARS-CoV-2 genomes belonging to other members of the *Coronaviridae* family and other common respiratory viruses were downloaded from the NCBI Virus database and ViralZone. Similar to inclusivity testing, 2423 non-SARS-CoV-2 representative unique genomes were obtained from the CD-HIT clustering, which were complemented with the human reference genome G1Kv37. These were consequently used as input in SCREENED for the 12 RT-qPCR tests with identical settings as the inclusivity testing, except for settings for the human genome that were less strict. The results are summarized in Table 4. For some assays, positive signals were obtained for genomes belonging to SARS-related coronaviruses and bat coronaviruses. As some primer and probe sets were intentionally designed to have a broad specificity to other coronaviruses in the context of a first sample screening (Table 1), the positive detection of these viruses with the corresponding sets was not counted as a false positive. An exclusivity of 100% was obtained for 26 primers and probe sets. For the other sets, an exclusivity of above 99% was determined for Assay_2_RdRp-P2 and Assay_10_N, and above 92% for Assay_10_E and Assay_12_E. Although these assays were expected to be strictly specific to SARS-CoV-2, they gave a theoretical positive RT-qPCR signal with SARS-related coronavirus and bat coronavirus. Nevertheless, the interpretation guidelines provided by the authors who developed Assays_10 (RdRp, S, E, and N) must be considered for the obtained results (Table 1). Indeed, for this RT-qPCR test, even though the respective primers and probe targeting the E and N genes can potentially detect SARS-related coronaviruses and/or bat coronaviruses, the other sets of the same test targeting the genes RdRp and S should remain negative, as was also confirmed with SCREENED (Table 4). In this situation, the overall final result of this RT-qPCR test will be “inconclusive”, and no false positive diagnosis will be given. For the human genome, no false positive identification was found with any of the primers and probe sets tested. 

Finally, it was observed that for Assay_2_RdRp-P2, Assay_10_E, and Assay_12_E, which did not show an exclusivity of 100%, one to two SNPs were present in the annealing sites of the primers and probes in the non-SARS-CoV-2 genomes that gave a positive signal in SCREENED. For Assay_10_N, there was a perfect match between the primers and non-SARS-CoV-2 genomes, leading to a positive signal (data not shown).

### 2.4. Evolution of the Inclusivity of Four RT-qPCR Tests after One Month

With the aim of evaluating the stability of the assay’s specificity over time, a second batch of SARS-CoV-2 genomes (1165), sequenced from samples collected between the 7th of April and the 7th of May 2020, was downloaded from the GISAID EpiCoV and NCBI Virus databases. After clearing this dataset from low-quality sequences and the clustering of identical genomes, 968 representative unique SARS-CoV-2 genomes were obtained. These genomes were then used in SCREENED for the inclusivity evaluation of Assays_1 (ORF1b and N) and Assays_8 (RdRp/Hel, S, and N) which had demonstrated the worst and best performance, respectively, when evaluated with the first batch of SARS-CoV-2 genomes (Section 2.2). Assays_2 (RdRp-P1, RdRp-P2, E, and N) and Assays_4 (N-1, N-2, and N-3) were also evaluated because they are some of the most widely used methods in Europe and the USA, respectively [28,42] (Table 5). As for the first evaluation (see Section 2.2), Assay_1_N exhibited a low inclusivity that had moreover decreased from 86.03% in the first batch to 74.54% in the second batch. Inspection revealed that this low score was again due to three substitutions, and even four in one case, in the beginning of the forward primer sequence. The other primers and probe sets evaluated with this more recent dataset all demonstrated a 100% inclusivity, except Assay_2_RdRp-P1, Assay_2_RdRp-P2, and Assay_8_N, which showed an inclusivity of between 99% and 100%. Interestingly, in a small number of genomes (4 or less), some new mismatches appeared in the region targeted by Assays_1_N, Assay_2_RdRp-P1, Assay_2_RdRp-P2, and Assay_8_N, while other mismatches previously detected during the first evaluation were not present anymore in the region targeted by Assay_1_ORF1b, Assay_1_N, Assay_2_N, and Assay_8_N. Contrarily, sequence alignment showed that the mismatches identified in Table 3 for Assay_2_RdRp-P1, Assay_2_RdRp-P2, and Assay_8_RdRp were still present in all the screened genomes (data not shown).

**Figure 4:**
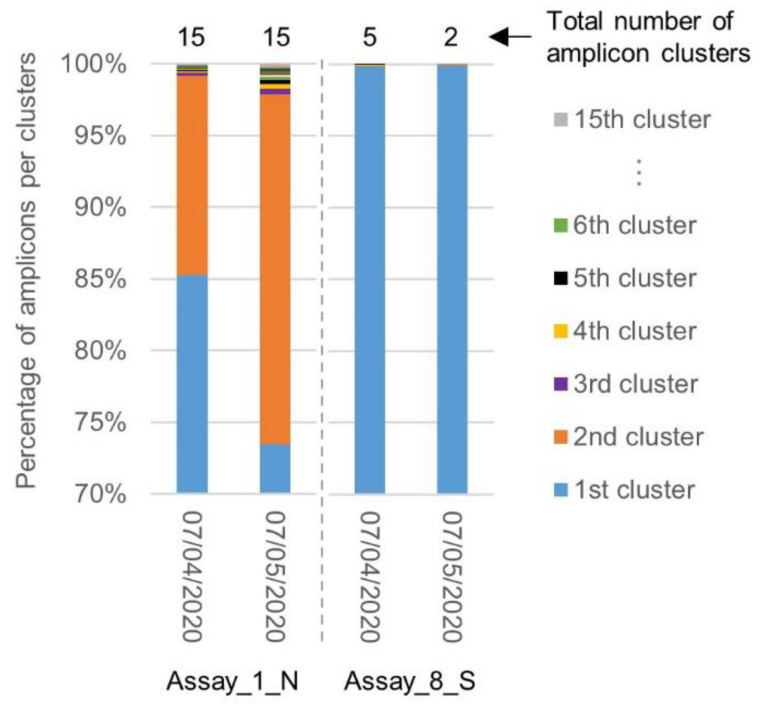
Comparison of amplicon diversity in the SARS-CoV-2 genomes collected before and after the 7th of April 2020 for Assay_1_N and Assay_8_S. The chart shows for Assay_1_N and Assay_8_S the repartition of the sequence amplified in the genomes downloaded before (2569) and after (968) the 7th of April, in their clusters. After one month, the diversity in the region targeted by Assay_1_N increased, while the region targeted by Assay_8_S stayed highly conserved.

Similar to the first inclusivity evaluation, a clustering was performed by SCREENED of the amplicons generated by the primers and probe sets of Assay_1_N, exhibiting the worst inclusivity associated with a large diversity in the targeted sequence, and Assay_8_S, exhibiting the best inclusivity associated with targeting a highly conserved region (Section 2.2). The diversity in the targeted sequences amplified by the corresponding primers was compared between both batches of SARS-CoV-2 genomes. While the total number of amplicon clusters generated for Assay_1_N remained unchanged, the percentage of amplicons contained in the second cluster increased. The region targeted by Assay_8_S remained, however, highly conserved, with one large cluster containing almost all amplicons (Figure 4). 

## 3. Discussion

When developing pathogen detection methods, such as RT-qPCR tests for SARS-CoV-2, it is of major importance to properly evaluate, amongst other criteria, the method’s specificity. This evaluation should be conducted when initially designing the diagnostic test and ideally be repeated periodically during the course of the pathogen’s spread in the population, especially for rapidly evolving pathogens. As this type of evaluation is time-intensive in the wet lab, or even infeasible through the absence of a representative strain collection, a first specificity assessment can be performed in silico with the use of publicly available whole genome sequencing data and appropriate bioinformatics tools. Now that thousands of SARS-CoV-2 genomes have been deposited in public repositories, in particular the GISAID database, the in silico inclusivity and exclusivity of 30 RT-qPCR primers and probe sets were evaluated in the current study using SCREENED, which allows extracting the number of theoretical false negative and false positive results from the alignment statistics.

The worst inclusivity was obtained for the primers and probe set of Assay_1_N, developed by the China CDC, mainly due to three substitutions present at the beginning of the forward primer annealing site. These substitutions were present in 358 unique genomes obtained mostly from SARS-CoV-2 strains isolated in European countries, as well as in USA/Canada and Australia, between the 25th of February 2020 and the 30th of March 2020. This indicates that these three mutations appeared probably at a later stage during the pandemic outside of China, and thus could not have been taken into account by the China CDC in developing Assay_1_N in January. The same mismatches were retained and even increased in frequency in new genomes collected between the 7th of April 2020 and the 7th of May 2020, indicating that they continue to spread in the viral population. Assay_1_N therefore does not meet the specific performance criteria elaborated by the EU commission [34], which were used to define the SCREENED settings in our analysis. The other evaluated primers and probe sets were more in agreement with these criteria, as their inclusivity was above 99% and only rarely were some limited mutations detected in primers and probe annealing regions. Interestingly, when the inclusivity of Assays 1 (ORF1b and N), Assays_2 (RdRp-P1, RdRp-P2, E, and N), Assays_4 (N-1, N-2, and N-3), and Assays_8 (RdRp/Hel, S, and N) was evaluated again with a new dataset of SARS-CoV-2 genomes downloaded one month after the first one; some of these SNPs were not detected any more, while few new ones were observed. We hypothesize that these are single events that did not spread largely, in contrast to the three substitutions present in many genomes isolated in European countries, USA/Canada, and Australia that increased over time. Concerning the diversity among the amplicons, the targeted sequences amplified by Assay_1_N, Assay_11_N-1, and Assay_11_N-2 showed a high number of variations in the targeted N locus, contrarily to the sequence amplified by Assay_8_S in the S locus, in which only a few SNPs were retrieved. These observations match with the genomic evolution of the virus determined since its first appearance in December 2019 (https://nextstrain.org/ncov/global?d=entropy&p=full, accessed June 24, 2020 [43]). Indeed, the loci targeted by Assay_1_N, Assay_11_N-1, and Assay_11_N-2 are in regions showing an important genetic evolution, while the region of the S gene targeted by Assay 8 is highly conserved. The future development of RT-qPCR assays targeting SARS-CoV-2 should take into account the genetic evolution of the virus, which is better known now, for the choice of highly conserved loci as targets.

In conclusion, for the inclusivity evaluation, except for Assay_1_N, all assays showed very few false negative results, in line with the findings of other similar studies [28,35,36,37,38]. A limited bias in our analysis can be due to the fact that genome data are typically generated from samples tested positive by RT-qPCR, such as the ones investigated in our study, or that sequencing primers identical to those included in the same diagnostic methods can be used. Nevertheless, the obtained good inclusivity results are more likely to be linked to the currently generally accepted moderate mutation rate of SARS-CoV-2, similar to SARS-CoV [22,44], and the fact that the virus has been spreading for only 6 months and is still genetically close to the original strain. However, the mutation rate of the virus could potentially increase in the future after the introduction of selection pressure induced by vaccines or treatments, as already demonstrated for other viruses, such as Influenzae [45]. To better appreciate any bias, it would be beneficial to add to the public databases enough relevant metadata with the exact information on how the SARS-CoV-2 positive samples were determined (including which RT-qPCR assay was used, if determined as such), and how the libraries for the WGS of the viral genome were made, as this information is not always available. For the latter, both open (metatranscriptomics) [22] as well as targeted approaches (PCR-based) [46,47,48] have been described in the literature. 

As for the exclusivity evaluation, which was not included in some of the previous similar studies [28,35,36,37,38], some SARS-related coronavirus and unclassified bat-coronavirus genomes gave a positive signal for some primers and probe sets, even though one to two SNPs were sometimes present in the annealing sites, for which the impact should be investigated in vitro. This positive signal is not surprising, as a close genomic similarity was observed between the first SARS-CoV and the bat-coronavirus suspected to be the origin of the COVID-19 zoonosis. Nevertheless, even though SARS-CoV has currently disappeared in the human population (no new cases since 2004 [49]), its reemergence remains a possibility, which consequently could result in false-positive SARS-CoV-2 detection with these not fully exclusive RT-qPCR assays. However, except for Assays_2 (RdRp-P1, RdRp-P2, E, and N) and Assay_12_E, the overall interpretation of assays—i.e., considering also the results of the other primers and probe set(s) that compose some assays—could avoid an incorrect diagnosis. Interestingly, for Assay_2_RdRp-P2, similar false-positive results as obtained in our in silico study were obtained in the wet lab by Chan and colleagues, who detected SARS-CoV when using the probe P2 targeting the RdRp gene that is considered strictly specific to SARS-CoV-2 [30]. This indicates that our in silico analysis can be backed up by in vitro data.

For Assay_2_RdRp-P1, Assay_2_RdRp-P2, Assay_5_N, Assay_8_RdRp, and Assay_10_E, some point mutations, not considered by the SCREENED criteria because 10% mismatches were allowed, were surprisingly identified in the totality, or almost the totality, of the screened SARS-CoV-2 genomes. Some of these SNPs, like those in the reverse primer and probe sequences of Assays_2 (RdRp-P1 and RdRp-P2), were deliberately introduced to allow the detection of both SARS-CoV-2 and other SARS-related coronaviruses, such as SARS-CoV and bat SARS-like coronaviruses. However, even though a low number of mismatches will not result in a total failure of the PCR reaction, a few mismatches can still potentially lower the sensitivity of the assay. The sensitivity of Assay_2_RdRp-P2 (Charité) was already demonstrated in the wet lab to be lower than that of other assays investigated in this study, and it was hypothesized that these SNPs present in almost all SARS-CoV-2 genomes could be the reason for this [28,30]. As the utmost sensitivity is required for SARS-CoV-2 detection, especially when the viral load is low depending on the time and nature of the sampling, it might be proposed to correct such mismatches with the aim to potentially increase the sensitivity of Assay_2_RdRp-P1, Assay_2_RdRp-P2, Assay_8_RdRp, and Assay_10_E. The SNP present in the reverse primer of Assay_5_N was already corrected in a revised version of the protocol but has not yet been updated in the WHO technical guidance [27,41]. Alternatively, to improve the limit of detection of diagnostic tests, other, although less commonly implemented nucleic acid detection technologies, such as droplet digital PCR (ddPCR), might be promising [50]. Some ddPCR tests were developed for SARS-CoV-2 detection and showed a higher sensitivity than RT-qPCR methods, which could be especially interesting for samples with low viral loads and/or containing RT-qPCR inhibitors [51,52,53], such as environmental samples to be used for potential future surveillance strategies [54]. As this technology is based on nucleic acid detection using specific pairs of primers, it will be interesting to evaluate their in silico specificity as well in the future.

The RT-qPCR test developed by Corman and colleagues at Charité (Berlin) is the most widely used in Europe [42,55]. This assay showed an overall good exclusivity, inclusivity, and stability over time, but the correction of some mismatches present in an abnormally high number of genomes could improve the sensitivity of this test, as elaborated on above. Considering all the parameters investigated in our study, the primers and probe set of Assay_8_S developed by Chan and colleagues [30] clearly showed the best results, taking the current variation of the virus into account. Indeed, this set was both 100% inclusive and exclusive, and targets a locus in the S gene that appears, until now, to be highly conserved and stable over time. The diversity in the amplicon sequences was very limited and remained unchanged when using a new dataset of SARS-CoV-2 genomes collected one month after the first one, indicating that this locus is a good RT-qPCR target, although this should be re-evaluated on regular basis. Because of its high specificity and low diversity, Assay_8_S could be used on its own without requiring another screening or confirmatory assay, even though it is true that the use of two molecular markers in a RT-qPCR assay is recommended, as it can lower the probability of incorrect results in case of drifting mutations in one of the targets. Considering this, Assay_8_S could be combined in one assay with Assay_9_ORF1a developed by Lu and colleagues [3], which also demonstrated excellent results in terms of specificity and low diversity. 

Our current results clearly demonstrate both the usefulness and requirement of in silico specificity evaluation, which was made possible on a several thousand genomes thanks to SCREENED. Here, this was performed retrospectively for already-existing RT-qPCR tests, but ideally this is already actively performed during the design and development of new diagnostic tests. Besides allowing the assessment of already developed RT-qPCR assays according to criteria proposed by EU guidelines, our analysis highlights that some virus mutations do have the potential to affect the sensitivity and specificity of RT-qPCR tests. Evidently, this was an in silico study that cannot account for the multitude of relevant in vitro parameters that also affect PCR-like reactions, nor can it replace the crucial process of validation using actual samples. Nevertheless, now that potentially impacting mismatches have been identified, their effect on the RT-qPCR outcome can be further investigated in the wet lab. Indeed, it would be interesting to compare for all the assays evaluated in our study the actual sensitivity obtained in the wet lab, as was for instance done by Vogels and colleagues [28] for four RT-qPCR tests, versus the inclusivity/exclusivity we observed with our in silico platform using several SARS-CoV-2 strains collected at different time points all over the world, including those with several mutations. Actually, it would be interesting to put effort into the harmonization of the validation of the SARS-CoV-2 RT-qPCR tests (e.g., reference material to share, performance criteria), as already initiated by the EU [34], in order to be able to properly compare the performance of the methods for certain parameters such as sensitivity. Moreover, to determine if new stable mutations will appear in SARS-CoV-2, potentially impacting the specificity of RT-qPCR tests, an evaluation similar to what was presented in this study should be conducted regularly and definitely as soon as a vaccine or treatment is in place, as new data are uploaded to the genomic databases daily.

## 4. Material and Methods

### 4.1. Collection of WGS Data

On the 7th of April 2020, a first batch of 3590 SARS-CoV-2 genomes were downloaded (batch 07042020): 3211 from the GISAID EpiCoV database (https://www.epicov.org, GISAID, Munich, Germany), 375 (including the reference strain NC_045512.2) from the NCBI Virus database (https://www.ncbi.nlm.nih.gov/labs/virus/, NCBI, Bethesda, MD, USA), and 4 from the China National GenBank (CNGB) hCoV-19 database (https://db.cngb.org/datamart/disease/DATAdis19, CNGBdb, Shenzhen, China). For the EpiCoV GISAID database, the settings “complete genomes” and “exclude low coverage genomes” were used. Genomes coming from the NCBI Virus database were complete sequences with “SARS-CoV-2” as the species (taxid: 2697049) and *Homo sapiens* as the “host” (taxid: 9606). For the CNGB, all the assemblies in the hCoV-19 database were included and were complete sequences. 

On the 23rd of April 2020, a batch of genomes belonging to other members of the *Coronaviridae* family (2549 including genomes from SARS-CoV, MERS-CoV, human coronavirus 229E, human coronavirus HKU1, human coronavirus NL63, batcoronavirus, and coronaviruses from more than 50 other host species) and to 75 other respiratory viruses (genomes from Adenovirus, Bocavirus, Influenza A(H1N1), Influenza A(H2N2), Influenza A(H3N2), Influenza A(H5N1), Influenza A(H7N9), Influenza A(H9N2), Influenza B, Metapneumovirus, Orthopneumovirus, Parainfluenza, and Rhinovirus/Enterovirus) were downloaded from the NCBI Virus database (only RefSeq data) and from ViralZone (https://viralzone.expasy.org, Swiss Institute of Bioinformatics, Geneva, Switzerland) (batch 23042020). The human genome reference Build HumanG1Kv37 provided by the Broad Institute (http://ftp.1000genomes.ebi.ac.uk/vol1/ftp/technical/reference/human_g1k_v37.fasta.gz, Broad Institute, Cambridge, MA, USA) was also included.

Finally, on the 7th of May 2020, a second batch of 1065 and 100 SARS-CoV-2 genomes were downloaded from the GISAID EpiCoV database and the NCBI Virus database, respectively (batch 07052020). These genomes were sequenced from samples collected after the 7th of April 2020. No new genomes were downloaded from the CNGB hCoV-19 database because none were added to this database since the 7th of April. 

### 4.2. Sequence Identity Clustering to Obtain Unique Representative Genomes

Sequence identity clustering was first performed for each batch of viral genomes for two reasons. Firstly, the genome sequence for the same sample can potentially be deposited in multiple databases that, however, all use different identifiers that cannot be reconciled. Secondly, the aim was to not bias the results by employing identical genome sequences multiple times (even if the genome sequence was obtained from different samples), which could artificially alter the inclusivity and/or exclusivity of evaluated tests. Consequently, all the downloaded genomes, separately for each of the three batches, were clustered with CD-HIT-EST (http://weizhong-lab.ucsd.edu/cdhit_suite/cgi-bin/index.cgi?cmd=cd-hit-est, University of California, San Diego, CA, USA [56]) using a sequence identity cut-off of 1.0 (other parameters were left at default settings) to group all the genomes with identical sequences into one cluster. Only the representative genome of every cluster was retained for further analysis. Finally, representative genomes of lower quality—i.e., showing more than three ambiguous nucleotides (such as “N”) in the genomic regions targeted by the evaluated RT-qPCR assays—were discarded. For batch 23042020, the human genome was not included in this clustering analysis because only one genome—i.e., the human reference genome—was employed. The final lists of employed unique representative genomes for batch 07042020, 07052020, and 23042020 are accessible in Supplementary file S1.

### 4.3. Settings and Input Files Used in SCREENED

SCREENED v1.0 [39] was employed for evaluating the RT-qPCR tests for each batch of unique representative genomes. This tool applies a two-step BLAST approach that first extracts, from the genome to be screened, the genomic region targeted by the RT-qPCR assay, and afterwards investigates the hybridization properties of the recovered region and the RT-qPCR primers and probe sets. SCREENED is available as an open-source tool that can be executed through a user-friendly interface using the Galaxy Workflow Management system [57] incorporated in the public Galaxy @Sciensano instance (https://galaxy.sciensano.be, Sciensano, Brussels, Belgium), or alternatively that can be downloaded (https://github.com/BioinformaticsPlatformWIV-ISP/SCREENED, GitHub, San-Francisco, CA, USA) to be run locally on the Linux command line.

As input, SCREENED requires a FASTA file containing all the genomes to be analyzed. In the present study, this corresponded to the three representative unique genomes batches obtained as described in Section 4.2, with one FASTA file per batch. A text file in tab-delimited format containing the sequences of the primers/probe sets and the reference templates for the targeted genomic region for every RT-qPCR method is also required [39] (Supplementary file S2). The employed sequences for primers and probes were taken from their corresponding publications/reports and are listed in Table 1. Reference templates were obtained by aligning the primer sequences from each RT-qPCR assay against the reference SARS-CoV-2 genome NC_045512.2 with MEGA X [58] (using MUSCLE and default settings) and extracting the amplicon sequence located between the two primers (with primer sequences included), and are listed in Supplementary file S2.

The following settings were used in SCREENED for inclusivity and exclusivity testing. All the investigated primers and probe sets were considered as resulting in a positive RT-qPCR signal when analyzing the screened genomes when (i) a maximum 10% mismatches were present in the annealing site of the screened genome for the primers and probe (irrespective of the alignment length); (ii) a minimum alignment length of 90% was observed in the annealing site of the screened genome for the primers and probe (irrespective of the total number of mismatches); and (iii) no single mismatch was present in the last five nucleotides at the 3′end for the forward and reverse primer [23,24,25,26]. The longest sequence amongst the oligonucleotides investigated in the current study is the forward primer of Assay_8_S, containing 30 nucleotides (Table 1). For this sequence, three mismatches (10% of 30) are tolerated for contributing to a theoretical positive signal (if the reverse primer and probe are also annealing correctly according to the SCREENED criteria). All the other primer and probe sequences were composed of less than 30 nucleotides so that no more than two mismatches were tolerated. These settings were selected in SCREENED in compliance with the EU criteria [34] for the performance evaluation of SARS-CoV-2 detection methods, stating that one to two mismatches can be tolerated for primers and probes with melting temperatures above 60 °C when used in a RT-qPCR reaction with an annealing temperature of 55 °C. Except for Assays_1 (ORF1b and N), Assays_10 (RdRp, S, E, and N), and Assays_11 (N-1, N-2, ORF1a-3, ORF1a-4, S-5, and S-6), for which this information was not communicated in their protocol, all other assays are performed with an annealing temperature equal or superior to 55 °C (Table 1). Both the amplicon and fragment extension options in SCREENED were enabled. Lastly, the clustering option of SCREENED was set to greedy to perform the clustering of all retrieved amplicons (i.e., the genomic regions targeted by the primers). From the clustered amplicons, the unique representative sequences were aligned with their related primers and probe sequences using MEGA X [58] (using MUSCLE and default settings).

For the human reference genome (batch 23042020), this procedure was adapted as follows. The input FASTA file contained the different (pseudo)chromosomes present in the HumanG1Kv37 build provided by the Broad Institute. The selection criteria of SCREENED were made less stringent by enforcing (i) a maximum of 30% allowed mismatches; (ii) a minimum alignment percentage of 70%; and (iii) allowing two mismatches in the last five bases at the 3′ end. This strategy was motivated by potentially allowing false positive identifications by employing looser settings that could then be investigated in more detail, but the absence of any positive signal confirmed perfect specificity for the human genome.

For all RT-qPCR assays using the SYBR Green technology, the above procedure was slightly adapted by using a mock sequence of 20 A nucleotides as the probe sequence in SCREENED (Supplementary file S2). Because of this, SCREENED labeled all the genomes as not resulting in a positive RT-qPCR signal. The detailed output files of SCREENED containing the results for all the selection criteria detailed above were then employed to filter the genomes for which both the forward and reverse primers annealed correctly (i.e., without considering the probe results). These filtered genomes were considered as resulting in a positive signal.

### 4.4. Determination of the In Silico Analytical Specificity of the Evaluated RT-qPCR Assays

The inclusivity was determined based on the SCREENED results obtained using all SARS-CoV-2 genomes by considering any SARS-CoV-2 genome not resulting in a theoretical RT-qPCR positive signal as a False Negative result (FN). The percentage of inclusivity for each primers and probe set was then calculated as follows (Equation (1)):

(1) $$Inclusivity\;\left(\%\right)=\left(1-\frac{Number\;of\;FN}{Total\;number\;of\;representative\;SARS-CoV-2\;genomes}\right)\times 100.$$

The exclusivity was determined based on SCREENED results obtained using all non-SARS-CoV-2 genomes by taking into account the recommendations given by the corresponding initial authors for the interpretation of their RT-qPCR test. Some RT-qPCR assays use primers and probe sets that are considered strictly specific to SARS-CoV-2, while others have a broader specificity to other *Sarbecovirus* species (Table 1). Consequently, if primers and probe sets, intended to have a wider specificity (see Section 2.1), were positive for other coronaviruses, they were not considered as False Positive results (FP). However, if the method is intended to be strictly specific to SARS-CoV-2, any theoretical positive signal against a non-SARS-CoV-2 genome was considered as FP. From this, the percentage of exclusivity of primers and probe sets targeting SARS-CoV-2 and *Sarbecovirus* was calculated with Equations (2) and (3), respectively:

(2) $$\;Exclusivity\;\left(\%\right)=\left(1-\frac{Number\;of\;FP}{Total\;number\;of\;non-SARS-CoV-2\;genomes}\right)\times 100,$$

(3) $$\;Exclusivity\;\left(\%\right)=\left(1-\frac{Number\;of\;FP}{Total\;number\;of\;non-\mathrm{Sarbecovirus}\;genomes}\right)\times 100.$$

## Figures and Tables

**Table 1:** Overview of the 12 RT-qPCR tests, each including one or several assays, investigated in this study.

Assay	Technology	Target	Primer and Probe Sequences (5′-3′)	Amplicon’s Starting Positio ^¶^	Amplicon Length	Annealing T °C	Intended Specificity	Guidelines for Overall Interpretation of the Assay	Source
1	TaqMan	ORF1b	Fw CCCTGTGGGTTTTACACTTAA	13,341	119	NC	NC *	NC^1^	China CDC, China [27]
			Rv ACGATTGTGCATCAGCTGA						
			P CCGTCTGCGGTATGTGGAAAGGTTATGG						
1	TaqMan	N	Fw GGGGAACTTCTCCTGCTAGAAT	28,880	99	NC	NC *		
			Rv CAGACATTTTGCTCTCAAGCTG						
			P TTGCTGCTGCTTGACAGATT						
2 **	TaqMan	RdRp-P1	Fw GTGARATGGTCATGTGTGGCGG	15,430	100	58 °C	*Sarbeco*	The E target can be used for a first screening. Then, positive results must be confirmed by RdRp sets, which must both be positive for the specific detection of SARS-CoV-2.	Charité Hospital, Germany [27]
			Rv CARATGTTAAASACACTATTAGCATA						
			P CCAGGTGGWACRTCATCMGGTGATGC						
2 **	TaqMan	RdRp-P2	Fw GTGARATGGTCATGTGTGGCGG	15,430	100	58 °C	SARS-		
			Rv CARATGTTAAASACACTATTAGCATA				CoV-2		
			P CAGGTGGAACCTCATCAGGAGATGC						
2 ***	TaqMan	E	Fw ACAGGTACGTTAATAGTTAATAGCGT	26,268	113	58 °C	*Sarbeco*		
			Rv ATATTGCAGCAGTACGCACACA						
			P ACACTAGCCATCCTTACTGCGCTTCG						
2 ****	TaqMan	N	Fw CACATTGGCACCCGCAATC	28,705	128	58 °C	NC		
			Rv GAGGAACGAGAAGAGGCTTG						
			P ACTTCCTCAAGGAACAACATTGCCA						
3	TaqMan	RdRp-IP2	Fw ATGAGCTTAGTCCTGTTG	12,689	108	58 °C	SARS-	RdRp-IP2 and RdRp-IP4 must be detected for SARS-CoV-2 determination. The E target can be used as a confirmatory result.	Institut Pasteur, France [27]
			Rv CTCCCTTTGTTGTGTTGT				CoV-2		
			P AGATGTCTTGTGCTGCCGGTA						
3	TaqMan	RdRp-IP4	Fw GGTAACTGGTATGATTTCG	14,079	107	58 °C	SARS-		
			Rv CTGGTCAAGGTTAATATAGG				CoV-2		
			P TCATACAAACCACGCCAGG						
3 ***	TaqMan	E	Fw ACAGGTACGTTAATAGTTAATAGCGT	26,268	113	58 °C	*Sarbeco*		
			Rv ATATTGCAGCAGTACGCACACA						
			P ACACTAGCCATCCTTACTGCGCTTCG						
4	TaqMan	N-1	Fw GACCCCAAAATCAGCGAAAT	28,286	72	55 °C	SARS-	Both N-1 and N-2 tests must be positive to confirm the detection of SARS-CoV-2. If only one target is detected, the result of the test is inconclusive.	US CDC, USA [27]
			Rv TCTGGTTACTGCCAGTTGAATCTG				CoV-2		
			P ACCCCGCATTACGTTTGGTGGACC						
4	TaqMan	N-2	Fw TTACAAACATTGGCCGCAAA	29,163	67	55 °C	SARS-		
			Rv GCGCGACATTCCGAAGAA				CoV-2		
			P ACAATTTGCCCCCAGCGCTTCAG						
4 ^†^	TaqMan	N-3	Fw GGGAGCCTTGAATACACCAAAA	28,680	72	55 °C	SARS-		
			Rv ACAATTTGCCCCCAGCGCTTCAG				CoV-2		
			P AYCACATTGGCACCCGCAATCCTG						
5	TaqMan	N	Fw AAATTTTGGGGACCAGGAAC	29,124	158	60 °C	SARS-	NA	NIID, Japan [27]
			Rv TGGCAGCTGTGTAGGTCAAC				CoV-2 ^§^		
			P ATGTCGCGCATTGGCATGGA						
6	TaqMan	ORF1b/	Fw TGGGGYTTTACRGGTAACCT	18,777	132	60 °C	*Sarbeco* ^‡^	The N gene detection is recommended for a first screening and the Orf1b/nsp14 detection as a confirmatory test. Mixed positive/negative results between the 2 targets should be regarded as undetermined.	HKU Med, Hong-Kong [27]
		nsp14	Rv AACRCGCTTAACAAAGCACTC						
			P TAGTTGTGATGCWATCATGACTAG						
6	TaqMan	N	Fw TAATCAGACAAGGAACTGATTA	29,144	110	60 °C	*Sarbeco* ^‡^		
			Rv CGAAGGTGTGACTTCCATG						
			P GCAAATTGTGCAATTTGCGG						
7	TaqMan	N	Fw CGTTTGGTGGACCCTCAGAT	28,319	57	55 °C	SARS-	NA	NIH, Thailand [27]
			Rv CCCCACTGCGTTCTCCATT				CoV-2 ^§^		
			P CAACTGGCAGTAACCA						
8	TaqMan	RdRp/	Fw CGCATACAGTCTTRCAGGCT	16,219	134	55 °C	SARS-	The primers and probe set targeting RdRp/Hel is the most sensitive of Assay 8 and can be used alone for the specific detection of SARS-CoV-2 with no cross-reactivity with other human coronaviruses.	Chan et al. [30]
		Hel	Rv GTGTGATGTTGAWATGACATGGTC				CoV-2		
			P TTAAGATGTGGTGCTTGCATACGTAGAC						
8	TaqMan	S	Fw CCTACTAAATTAAATGATCTCTGCTTTACT	22,711	158	55 °C	SARS-		
			Rv CAAGCTATAACGCAGCCTGTA				CoV-2		
			P CGCTCCAGGGCAAACTGGAAAG						
8	TaqMan	N	Fw GCGTTCTTCGGAATGTCG	29,209	97	55 °C	SARS-		
			Rv TTGGATCTTTGTCATCCAATTTG				CoV-2		
			P AACGTGGTTGACCTACACAGST						
9	TaqMan	ORF1a	Fw AGAAGATTGGTTAGATGATGATAGT	3192	118	58 °C	SARS-	NA	Lu et al. [3]
			Rv TTCCATCTCTAATTGAGGTTGAACC				CoV-2		
			P TCCTCACTGCCGTCTTGTTGACCA						
10	SYBR	RdRP	Fw CATGTGTGGCGGTTCACTAT	15,440	118	NC	SARS-	The main objective of this assay was the determination of patients negative for SARS-CoV-2. Thus, for a negative result, the 4 targets must remain undetected. Presence of SARS-CoV-2 is suspected if at least one target is detected, but further investigations need to confirm this.	Won et al. [31]
	Green		Rv TGCATTAACATTGGCCGTGA				CoV-2		
10	SYBR	S	Fw CTACATGCACCAGCAACTGT	23,113	100	NC	SARS-		
	Green		Rv CACCTGTGCCTGTTAAACCA				CoV-2		
10	SYBR	E	Fw TTCGGAAGAGACAGGTACGTT	26,258	107	NC	SARS-		
	Green		Rv CACACAATCGATGCGCAGTA				CoV-2		
10	SYBR	N	Fw CAATGCTGCAATCGTGCTAC	28,731	118	NC	SARS-		
	Green		Rv GTTGCGACTACGTGATGAGG				CoV-2		
11 ^∥^	SYBR	N-1	Fw GCCTCTTCTCGTTCCTCATCAC	28,816	111	NC	SARS	N and ORF1a targets can be detected in SARS-CoV-2 and possibly in SARS-CoV even if there are 2 SNPs of difference. S targets should be detected in SARS-CoV-2 only, but some variants can be missed. Consequently, a combination of these targets must be used for test development.	Sigma-Aldrich [32]
	Green		Rv AGCAGCATCACCGCCATTG						
11 ^∥^	SYBR	N-2	Fw AGCCTCTTCTCGTTCCTCATCAC	28,815	102	NC	SARS		
	Green		Rv CCGCCATTGCCAGCCATTC						
11 ^∥^	TaqMan	ORF1a-3	Fw CCGCAAGGTTCTTCTTCGTAAG	618	146	NC	SARS		
			Rv TGCTATGTTTAGTGTTCCAGTTTTC						
			P AAGGATCAGTGCCAAGCTCGTCGCC						
11 ^∥^	TaqMan	ORF1a-4	Fw GGCTTACCGCAAGGTTCTTC	612	152	NC	SARS		
			Rv TGCTATGTTTAGTGTTCCAGTTTTC						
			P AAGGATCAGTGCCAAGCTCGTCGCC						
11 ^∥^	TaqMan	S-5	Fw CAGGTATATGCGCTAGTTATCAGAC	23,564	97	NC	SARS-		
			Rv CCAAGTGACATAGTGTAGGCAATG				CoV-2		
			P AGACTAATTCTCCTCGGCGGGCACG						
11 ^∥^	TaqMan	S-6	Fw GCAGGTATATGCGCTAGTTATCAG	23,563	187	NC	SARS-		
			Rv ACACTGGTAGAATTTCTGTGGTAAC				CoV-2		
			P AGACTAATTCTCCTCGGCGGGCACG						
12	TaqMan	E	Fw ACTTCTTTTTCTTGCTTTCGTGGT	26,294	82	60 °C	SARS-	NA	Huang et al. [33]
			Rv GCAGCAGTACGCACACAATC				CoV-2		
			P CTAGTTACACTAGCCATCCTTACTGC						

E: envelope protein; Hel: helicase; N: nucleocapsid protein; nsp-14: non-structural protein 14; ORF1: Open Reading Frame 1; RdRp: RNA-dependent RNA polymerase; S: spiking protein. Fw: forward primer sequence; Rv: reverse primer sequence; P: probe sequence. NC: Not Communicated; NA: Not Adapted because assay is single target. SARS: SARS-CoV-2 and possibly SARS-CoV; *Sarbeco*: *Sarbecovirus*—i.e., SARS-CoV, SARS-CoV-2, and SARS-related coronaviruses. CDC: Center for Disease Control and prevention. NIID: National Institute for Infectious Diseases. NIH: National Institute for Health. Assays_*number* (*target list*): all the targets of a RT-qPCR test developed by an institute or a scientific team. Assay_*number*_*target*: one specific target of a RT-qPCR test developed by an institute or a scientific team. *: Assays_1 (ORF1b and N) was developed for SARS-CoV-2 detection but the authors of these assays did not define the intended specificity of each target included in it. **: Assay_2_RdRp-P1 and Assay_2_RdRp-P2 are using the same primers but with different probes. ***: Assay_2_E and Assay_3_E are using the same primers and probe sets for the detection of the E gene. ****: The N target was less investigated by the authors of Assays_2 (RdRp-P1, RdRp-P2, E, and N) because it was less sensitive than the other targets. Thus, the RT-qPCR test from Charité Hospital (Germany) can be performed with RdRp and E detection only. †: The third N target was present in the first version (February) of Assays_4 (N-1, N-2 and N-3) but was removed in a revised version (March). Thus, the US CDC RT-qPCR test can be performed with N-1 and N-2 detection only. ‡: Even though Assays_6 (Orf1b/nsp14 and N) are indicated to be specific to *Sarbecovirus*, the authors mentioned that they were developed for SARS-CoV-2 detection, as SARS-CoV is supposedly eradicated. §: The intended specificity was not clearly stated in the original protocol, but these assays were developed for SARS-CoV-2 detection. ∥: Primers and probes of Assays_11 (N-1, N-2, ORF1a-3, ORF1a-4, S-5, and S-6) were designed by Sigma-Aldrich to help the competent authorities in the diagnosis of COVID-19. However, to the best of our knowledge, no commercial kit has been developed yet. ^¶^: Positioning based on reference genome NC_045512.2.

**Table 2:** Inclusivity evaluation of primers and probe sets using SCREENED with the first SARS-CoV-2 genome batch 07042020 for all the RT-qPCR tests.

Assay	Target	Genomes with Mismatches in the First Five Nucleotides of the Primer’s 3′ End		Genomes with >10% Mismatches in the Annealing Sites of Primers and Probes		False Negative Results ***	Inclusivity
		Number *	Modifications **	Number *	Modifications **		
1	ORF1b	1	Fw GTGGGTTTTACA**T**TTAA	0	-	1	99.96%
1	N	1	Rv CAGACATTTTGCTCTCAA**A**CTG	358	Fw **AAC**GAACTTCTCCTGCTAGAAT	359	86.03%
2	RdRp-P1	0	-	1	Pb CCAGGTGG**G**AC**C**TCATCAGG**A**GATGC	1	99.96%
2	RdRp-P2	0	-	0	-	0	100%
2	E	0	-	0	-	0	100%
2	N	4	Fw CACATTGGCACCCG**T**AATC	0	-	5	99.81%
		1	Rv GAGGAACGAGAAGAG**A**CTTG				
3	RdRp-IP2	3	Rv CTCCCTTTGTTGTGTT**A**T	0	-	3	99.88%
3	RdRp-IP4	0	-	0	-	0	100%
3	E	0	-	0	-	0	100%
4	N-1	7	Rv TCTGGTTACTGCCAGTTGAA**C**CTG	0	-	7	99.73%
4	N-2	1	Fw TTACAAACATTGGCC**T**CAAA	0	-	1	99.96%
4	N-3	0	-	0	-	0	100%
5	N	3	Fw AAATTTTGGGGACCA**T**GAAC	0	-	8	99.69%
		4	Fw AAATTTTGGGGACCAGGAA**T**				
		1	Rv TGGCA**C**CTGTGTAGGT**A**AAC				
6	ORF1b/nsp14	0	-	0	-	0	100%
6	N	2	Rv CGAAGGTGTGACTTC**A**ATG	0	-	2	99.92%
7	N	7	Fw CGTTTGGTGGACCCTCAG**G**T	0	-	7	99.73%
8	RdRp/Hel	0	-	0	-	0	100%
8	S	0	-	0	-	0	100%
8	N	1	Fw GCGTTCTTCGGAATGTC**T**	0	-	1	99.96%
9	ORF1a	0	-	0	-	0	100%
10	RdRp	8	Rv TGCATTAACATTGGCCGT**A**A	0	-	8	99.69%
10	S	0	-	0	-	0	100%
10	E	0	-	0	-	0	100%
10	N	1	Rv GTTGCGACTACGTGATGAG**T**	0	-	1	99.96%
11	N-1	3	Fw GCCTCTTCTCGTTCCTCA**C**CAC	0	-	8	99.69%
		5	FW GCCTCTTCTCGTTCCTCAT**T**AC				
11	N-2	3	Fw AGCCTCTTCTCGTTCCTCA**C**CAC	0	-	8	99.69%
		5	Fw AGCCTCTTCTCGTTCCTCAT**T**AC				
11	ORF1a-3	0	-	0	-	0	100%
11	ORF1a-4	0	-	0	-	0	100%
11	S-5	9	Fw CAGGTATATG**T**GCTAGTTATCA**C**AC	0	-	11	99.57%
		1	Fw CAGGTATATGCGCTAGTTATCA**T**AC				
		1	Fw CAGGTATATGCGCTAGTTATC**G**GAC				
11	S-6	9	Fw GCAGGTATATG**T**GCTAGTTATCA**C**	0	-	11	99.57%
		1	Fw GCAGGTATATGCGCTAGTTATCG**T**				
		1	Fw GCAGGTATATGCGCTAGTTATC**G**G				
12	E	0	-	0	-	0	100%

-: No modifications retrieved by SCREENED. *: Number of genomes having the type of mismatches investigated from a total of 2569 unique representative genomes. **: Detected mismatches are indicated in bold in the 5′-3′ sequence of forward primers (Fw), reverse primers (Rv), and probes (Pb). The mismatch in bold, and underlined is an additional SNP not specific to the parameters investigated by SCREENED. ***: The total number of genomes that did not produce a positive signal for the evaluated primers and probe sets considered as false negative results. No false negative results were obtained due to minimum alignment length lower than 90%.

**Table 3:** SNPs present in an abnormally large number of SARS-CoV-2 genomes.

Assay	Target	Modifications *	Number of Genomes with the Modification(s)
2	RdRp-P1	Pb CCAGGTGGAAC**C**TCATCAGG**A**GATGC	2566 (99.88%)
2	RdRp-P1&P2	Rv CAAATGTTAAA**A**ACACTATTAGCATA	2569 (100%)
5	N	Rv TGGCA**C**CTGTGTAGGTCAAC ^†^	2569 (100%)
8	RdRp/Hel	Rv **A**TGTGATGTTGATATGACATGGTC	2569 (100%)
10	E	Rv CACACAATCGA**A**GCGCAGTA	2567 (99.92%)

*: Mismatches are indicated in bold in the 5′-3′ sequence of reverse primers and probe (Pb). These mismatches were determined by multiple alignment and not by SCREENED because these mismatches are not considered to prevent amplification, but potentially can result in false negative signals if secondary mismatches appear. ^†^: The reverse primer of Assay_5_N was corrected in a revised version of the protocol [41], but is not yet included in the WHO guidance document.

**Table 4:** Exclusivity evaluation of primers and probe sets using SCREENED with the non-SARS-CoV-2 genome batch 23042020 for all the RT-qPCR tests.

Assay	Target	Genomes Giving a Positive Signal	False Positive Results *	Exclusivity
1	ORF1b	0	0	100%
1	N	0	0	100%
2	RdRp-P1	172 SARS-related coronavirus	0	100%
2	RdRp-P2	5 SARS-related coronavirus and 2 unclassified bat coronavirus	7	99.75%
2	E	179 SARS-related coronavirus and 6 unclassified bat coronavirus	0	100%
2	N	162 SARS-related coronavirus and 5 unclassified bat coronavirus	0	100%
3	RdRp-IP2	0	0	100%
3	RdRp-IP4	0	0	100%
3	E	179 SARS-related coronavirus and 6 unclassified bat coronavirus	0	100%
4	N-1	0	0	100%
4	N-2	0	0	100%
4	N-3	13 SARS-related coronavirus and 1 unclassified bat coronavirus	0	100%
5	N	0	0	100%
6	ORF1b/nsp14	170 SARS-related coronavirus and 5 unclassified bat coronavirus	0	100%
6	N	179 SARS-related coronavirus and 5 unclassified bat coronavirus	0	100%
7	N	0	0	100%
8	RdRp/Hel	0	0	100%
8	S	0	0	100%
8	N	0	0	100%
9	ORF1a	0	0	100%
10	RdRP	0	0	100%
10	S	0	0	100%
10	E	181 SARS-related coronavirus and 5 unclassified bat coronavirus	186	92.32%
10	N	2 SARS-related coronavirus	2	99.92%
11	N-1	0	0	100%
11	N-2	0	0	100%
11	ORF1a-3	2 SARS-related coronavirus	2	100%
11	ORF1a-4	0	0	100%
11	S-5	0	0	100%
11	S-6	0	0	100%
12	E	178 SARS-related coronavirus and 6 unclassified bat coronavirus	184	92.41%

*: SARS-related coronavirus and bat-coronavirus were not counted as false positive results if detected by primer and probe sets designed to have a broad specificity to other members of the *Coronaviridae* family (see Table 2). No false positive results were obtained due to a minimum alignment length lower than 90%.

**Table 5:** Inclusivity evolution of the four RT-qPCR assays analyzed with SCREENED using the second batch of SARS-CoV-2 genomes.

Assay	Target	Genomes with Mismatches in the First Five Nucleotides of the Primer’s 3′ End		Genomes with too Many Mismatches in the Annealing Sites of Primers and Probes		False Negative Results ***	Inclusivity
		Number *	Modifications **	Number *	Modifications **		
1	ORF1b	0	-	0	-	0	100%
1	N	1	Fw GGGGAACTTCTCCTGCTA**A**AAT	241	Fw **AAC**GAACTTCTCCTGCTAGAAT	247	74.54%
		4	Fw GGGGAACTTCTCCTGCTA**C**AAT	1	Fw **AAC**GAACTTCTCCT**T**CTAGAAT		
2 ****	RdRp-P1	1	Fw GTGAAATGGTCATGTGT**A**GCGG	0	-	2	99.79%
		1	Fw GTGAAATGGTCATGTGTGG**T**GG	0	-		
2 ****	RdRp-P2	1	Fw GTGAAATGGTCATGTGT**A**GCGG	0	-	2	99.79%
		1	Fw GTGAAATGGTCATGTGTGG**T**GG	0	-		
2	E	0	-	0	-	0	100%
2	N	0	-	0	-	0	100%
4	N-1	0	-	0	-	0	100%
4	N-2	0	-	0	-	0	100%
4	N-3	0	-	0	-	0	100%
8	RdRp/Hel	0	-	0	-	0	100%
8	S	0	-	0	-	0	100%
8	N	1	Rv TTGGATCTTTGTCATCCAATTT**A**	0	-	1	99.90%

-: No modifications retrieved by SCREENED. *: Number of genomes exhibiting the type of mismatches investigated. **: Mismatches are indicated in bold in the 5′-3′ sequence of forward primers (Fw), reverse primers (Rv), and probes (Pb). ***: The total number of genomes which did not produce a positive signal for the evaluated primers and probe sets, according to SCREENED, were determined as false negative results. No false negative results were obtained due to a minimum alignment length lower than 90%. ****: The primers targeting the RdRp gene for Assay_2_RdRp-P1 and Assay_2_RdRp-P2 are identical, but the probe is different (not indicated in this table).

## Supplementary Materials

Supplementary materials can be found at https://www.mdpi.com/1422-0067/21/15/5585/s1.
